# Targeting Cdk5 for killing of breast cancer cells via perturbation of redox homeostasis

**DOI:** 10.18632/oncoscience.431

**Published:** 2018-06-27

**Authors:** Saranya NavaneethaKrishnan, Jesusa L. Rosales, Ki-Young Lee

**Affiliations:** Department of Cell Biology and Anatomy, Arnie Charbonneau Cancer Institute, University of Calgary, Calgary, AB, Canada

**Keywords:** breast cancer, phosphorylation, protein kinase, Cdk5, ROS

Reactive oxygen species (ROS) are highly reactive oxygen-containing molecules such as superoxide, hydroxyl radical, and H_2_O_2_. ROS are mainly produced as by-products during oxidative phosphorylation in mitochondria. Electron transport chain complex I generates superoxide ions toward the matrix side whereas complex III generates superoxide ions toward both the matrix and inter-membrane space. Superoxide ions are then converted into hydrogen peroxide (H_2_O_2_) by superoxide dismutase (SOD) and subsequently transformed into H_2_O by glutathione peroxidase. ROS play a key role in regulating numerous cellular processes, including cell growth and differentiation, phagocytosis and cell death. Thus, cellular ROS level is tightly controlled by ‘redox homeostasis’, which achieves balance by controlling ROS level through elimination via anti-oxidants [1]. Perturbation of redox homeostasis towards excess ROS brings about serious consequences such as ROS induced-irreversible oxidative damage to cellular building blocks that could trigger cell death [2]. Interestingly, it has been shown that rapidly growing and metabolically altered cancer cells have increased ROS levels compared to adjacent normal cells [1], putting cancer cells at a higher risk of reaching the threshold for ROS to instigate cell death, and thus, the implication that induction of further ROS insult in cancer cells may be utilized to kill cancer cells [1].

**Figure 1:**
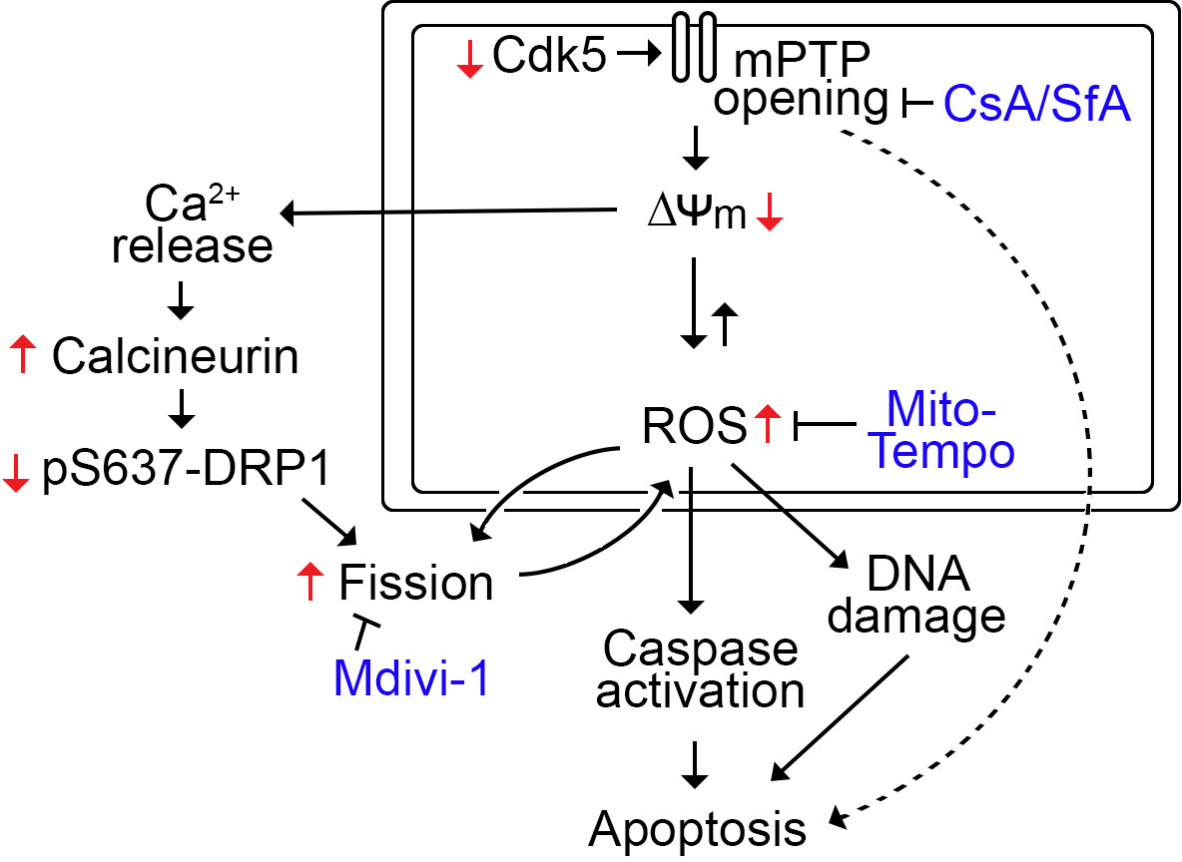
Molecular mechanism by which Cdk5 loss in breast cancer cells mediates mPTP opening and apoptosis Cdk5 loss triggers mPTP opening and subsequent mitochondrial depolarization and ROS increase, leading to the caspase activation and cell death. mPTP opening and mitochondrial depolarization also causes calcium release and calcineurin activation, which leads to dephosphorylation of DRP1 at S_637_, resulting in mitochondrial fission. ROS partially induce fission, which in turn, induce further ROS increase. In addition, ROS increase causes overwhelming DNA damage that leads to cell death.

Our previous analysis of publicly available multiple microarray datasets that measured levels of cyclin-dependent kinase 5 (Cdk5) in cancer vs corresponding normal tissues showed considerable upregulation of Cdk5 level in breast cancer [3]. Recently, we found that loss of Cdk5 in breast cancer cells causes intracellular ROS increase [4], suggesting that Cdk5 could potentially be an ideal target for triggering ROS increase in breast cancer cells. Specifically, we demonstrated that Cdk5 loss in the ER+ MCF-7, HER2+ SKBR3 and triple negative MDA MB-231 breast cancer cells causes ROS increase in both mitochondria and cytoplasm and this is accompanied by increased cell death [4]. ROS increase in Cdk5-depleted breast cancer cells coincided with mitochondrial depolarization and fragmentation. As these events are inter-dependent, we investigated the sequence of events following Cdk5 depletion in breast cancer cells. Since prolonged opening of the mitochondrial permeability transition pore (mPTP) could elicit mitochondrial depolarization or reduced membrane potential and ROS production that could lead to cell death, we examined the effect of the mPTP inhibitors, cyclosporine A (CsA) and sanglifehrin A (SfA), in breast cancer cells depleted of Cdk5 (Figure 1). We found that treatment with mPTP inhibitors reversed Cdk5 loss-mediated phenotypes (e.g., ROS increase, mitochondrial depolarization and cell death), suggesting that mPTP opening in the inner mitochondrial membrane occurs prior to mitochondrial depolarization and ROS increase. Treatment with Mito-Tempo, a mitochondrial ROS scavenger, partially restored mitochondrial membrane potential and reduced fragmentation (Figure 1), suggesting that increased ROS level partially accounts for mitochondrial depolarization and fragmentation in cells lacking Cdk5.

As supported by our findings, mPTP opening affects mitochondrial fusion and fission dynamics, which is essential for proper mitochondrial function. Dynamin-related protein 1 (Drp1) translocation into mitochondria and oligomerization at the site of fission, which causes membrane ingression, is an initial event that leads to mitochondrial fission, which is induced by elevated intracellular calcium ([Ca^2+^]_i_) [4]. In breast cancer cells, we observed that Cdk5 loss causes a rise in [Ca^2+^]_i_ that coincides with increased activity of the Ca^2+^-dependent calcineurin, and subsequent DRP1 at S_637_ dephosphorylation that stimulates mitochondrial fission [4]. While Cdk5 phosphorylation of Drp1 at Ser_616_ promotes fission in brain tumor initiating cells [5], the fact that CsA and SfA treatment reverses dephosphorylation of DRP1 S_637_ but not Ser_616_ suggests that in breast cancer cells, Cdk5 loss promotes mitochondrial fission through DRP1 S_637_ dephosphorylation.

Cytochrome C release and subsequent caspase activation and cell death could also result from prolonged mPTP opening and, indeed, cells depleted of Cdk5 showed increased activation of caspase-3 and -9 and apoptosis. Since apoptosis induced by Cdk5 loss is completely reversed by CsA and SfA and close to be fully inhibited by Mito-Tempo but unaffected by the Mdivi-1 inhibitor of mitochondrial fission, it appears that apoptosis is mediated via an mPTP-dependent mechanism and primarily through ROS increase (Figure 1).

Previous studies have indicated that loss of Cdk5 increases cancer cell sensitivity to chemotherapeutic drugs such as cisplatin and camptothecin as well as poly-ADP ribose polymerase (PARP) inhibitors [6] and bortezomib [7]. However, the molecular mechanism by which Cdk5 loss increases drug sensitivity and cell death, particularly in breast cancer cells, remains elusive. Our studies demonstrate a Cdk5-controlled mPTP machinery that maintains the functional integrity of mitochondria and modulates the intrinsic apoptotic pathway in breast cancer cells. It is possible that loss of Cdk5 in breast cancer cells causes increased susceptibility to drug therapy by deregulation of mPTP-mediated mitochondrial functions and synergistic effects of Cdk5 loss and drug therapy through ROS-induced overwhelming DNA damage and apoptosis. It would be interesting to know how Cdk5 inhibits opening of mPTP, a voltage-dependent and high-conductance channel that consists of cyclophilin D and F_0_-F_1_ ATP synthase [8].
